# Enhanced biological removal of intermittent VOCs and deciphering the roles of sodium alginate and polyvinyl alcohol in biofilm formation

**DOI:** 10.1371/journal.pone.0217401

**Published:** 2019-05-22

**Authors:** Rongfang Feng, Gang Zhao, Yonggang Yang, Meiying Xu, Shaobin Huang, Guoping Sun, Jun Guo, Jianjun Li

**Affiliations:** 1 School of Bioscience and Bioengineering, South China University of Technology, Guangzhou, PR China; 2 Guangdong Institute of Microbiology, Guangzhou, PR China; 3 State Key Laboratory of Applied Microbiology Southern China, Guangzhou, PR China; 4 Guangdong Provincial Key Laboratory of Microbial Culture Collection and Application, Guangzhou, PR China; 5 School of Environment and Energy, South China University of Technology, Guangzhou, PR China; CAS, CHINA

## Abstract

Developing a robust biofilm is a prerequisite for a biotrickling filter to obtain the good performance in removing volatile organic compounds (VOCs). But the biofilm formation can be seriously disturbed under intermittent loading condition due to carbon starvation stress in idle time. In this study, a biotrickling filter, with its packing materials being modified by 3% sodium alginate and 5% polyvinyl alcohol (v/v = 1:3), was employed to treat intermittent VOCs. Results showed that the removal efficiencies of toluene, ethylbenzene, *p*-xylene, *m*-xylene, and *o*-xylene was significantly enhanced in the BTF compared to the control one. Under relatively lower inlet loading, nearly complete removal of the five pollutants was achieved. A quantitative analysis showed that the concentration of total organic compound (TOC) in the leachate maintained at a high level, and had a strongly positive correlation with the divergence of microbial communities. The capacity of biofilm formation in the BTF was approximately four-fold higher than the control BTF, while the quantity of EPS secreted was more than ten-fold. EPS comprised largely of protein, and to less extent, polysaccharide. The biofilm formed on the modified packing materials maintained higher levels of microbial diversity and stability, even when modifiers were complete depleted or the VOCs inlet loading was increased. This study highlights the importance of packing materials for reducing the gap in performance between laboratory and industrial applications of BTFs.

## Introduction

Volatile organic compounds (VOCs) removal from waste gas with the use of biotrickling filters (BTFs) has received increasing attention because of its relative economy, high efficiency and stability [1–2]. These advantages are however only evident under relatively ideal conditions, such as a constant substrate supply and regular nutrient replenishment. In practice, industrial manufacturing releases VOCs intermittently due to shutdown at night and weekends or equipment maintenance. Qi and Moe (2006) reported significantly compromised performance of an intermittently-loaded biofilter compared to a continuous biofilter [3]. Similarly, other studies have suggested that the performance of BTFs in industrial application has been far lower than those used in laboratories [4].

The removal efficiency of VOCs is largely dependent on the activities of a number of diverse microorganisms that use VOCs as sole carbon or energy source to inhabit and proliferate within bioreactors and ultimately form a mature biofilm on the surface of packing materials. Intermittent VOCs supply disrupts biofilm formation by introducing frequent starvation stress [5]. Although this stress has been identified and reported [6–9], most BTFs currently in use continue to undergo acclimation under a continuous mode for rapid biofilm formation. However, how starvation stress resulting from intermittent VOCs supply results in a disruption of BTF construction is unclear, from initial acclimation (i.e. biofilm formation) to subsequent removal performance.

A key challenge for the treatment of intermittently loaded VOCs by BTFs is the establishment of a robust biofilm composed of diverse microorganisms. Biofilm describes a complex group of microbial cells which are embedded in slimy extracellular polymeric substances (EPS) [10]. EPS is crucial for biofilm maturation as it: 1) provides a scaffold for biofilm’s three-dimensional (3-D) architecture [11, 12]; 2) shields cells from extreme conditions [13] and; 3) serves as a carbon or energy source for microbial cells under nutrient-poor conditions [14]. Although solutions to this challenge have rarely been reported to date, it is evident that enhanced EPS secretion facilitates biofilm formation and maturation, which consequently boosts biodiversity and enhances VOCs removal.

The formation of biofilm can be divided into five stages: 1) reversible attachment of cells onto surfaces; 2) irreversible adhesion between cells and surfaces facilitated by the secretion of EPS; 3) surface colonization into microcolonies; 4) biofilm formation and maturation with a 3-D structure and; 5) the spread of biofilm to nearby surfaces [15]. Stage II determines the success of biofilm formation. EPS secretion also constitutes a limiting step since it is not triggered unless sufficient cells are adhered [12]. Cells under starvation stress are unable to firmly adhere to a surface, reducing the probability of the initiation of EPS secretion and preventing the transformation of reversible adhesion to irreversible adhesion. Sodium alginate and polyvinyl alcohol are commonly used for microbial embedding [16, 17] to manually accelerate Stage II and III to shorten acclimation. In addition, sodium alginate is characterized by unique colloid properties and can be degraded by bacteria [18]. Therefore, sodium alginate can act as a supplementary carbon source during periods of VOCs unavailability.

Given the above arguments, this study was conducted with the following three objectives: 1) to unravel whether and to what extent the removal of intermittent VOCs can be enhanced by the modified packing materials; 2) to decipher the roles of sodium alginate and polyvinyl alcohol in biofilm formation; 3) to explore the basic mechanisms that underlie microbial community succession.

## Materials and methods

### Modification of polyurethane foam

Polyurethane foam used in this study was purchased from Rong Ze environmental companies (Jiangsu, China), with specific surface area of 13.2 m^2^ g^-1^, bulk density of 8.6 kg m^-3^, porosity of 97.78%. Polyurethane foam was cut into 1 cm × 1 cm × 1 cm cubes, immersed into modifiers (a 1:3 mixture of 3% sodium alginate and 5% polyvinyl alcohol) and extruded using tweezers to remove bubbles. Once the cubes were completely encased with the modifiers, they were transferred into a 1% CaCl_2_ solution. After 10 min, the cubes were soaked in water for 30 min for subsequent use in the treatment. For the control, the commercial polyurethane foam cubes of the same dimensions were immersed in water for 30 min. Bulk density of the modified polyurethane foam increased up to 35.65 kg m^-3^, and the water holding rate increased to 27.14% (with 18.40% of the control polyurethane foam).

### Source of the microbial consortia

Three microbial consortia were used as inoculum for the startup of the two BTFs, which had been acclimatized for more than two years with toluene, xylene and styrene as sole carbon source, respectively. Specific acclimation procedure was described in our previous work [19]. Before been inoculated, each consortium was inoculated into 500 mL mineral medium containing glucose at 0.5% (m/v), and shook for two days at 180 rpm, 30°C. Subsequently, the microbial solutions were centrifuged for 10 min at 8,000 g. Resulted pellets were collected and washed twice with phosphate buffer solution (NaCl 8 g L^−1^, KCl 0.2 g L^−1^, Na_2_HPO_4_ 1.42 g L^−1^, KH_2_PO_4_ 0.27 g L^−1^), and then were resuspended by mineral medium. The three microbial solutions were mixed and inoculated into the two BTFs equally. The mineral medium was comprised of NH_4_Cl (2.5 g L^−1^), Na_2_HPO_4_ (1.0 g L^−1^), KH_2_PO_4_ (0.7 g L^−1^), MgSO_4_ (0.05 g L^−1^) and CaCl_2_ (0.015 g L^−1^).

### BTF setup and operation

Two identical laboratory-scale BTFs employed in the present study were made of plexiglass. Both BTF1 and BTF2 were comprised of two columns, with an inner diameter of 10 cm and a total height of 38 cm. The control polyurethane foam cubes were packed into BTF1 (control) whereas the modified cubes were packed in BTF2 (treatment). The VOCs solution (toluene, ethylbenzene, *p*-xylene, *m*-xylene and *o*-xylene) was mixed with air in a chamber and loaded into the BTFs from the bottom.

Both BTFs were operated under an intermittent mode (8 hours per day, 5 days per week) in parallel with an 80-day duration. During non-operational periods, BTFs were supplied with VOCs-free air and a nutrient solution with the same flow velocity as during the operational periods. The empty bed residence time (EBRT) was set to 60 s. The nutrient solution was renewed approximately once every two weeks to ensure sufficient nutrients and moisture for microbial growth. The inlet concentrations of toluene, ethylbenzene, *p*-xylene, *m*-xylene and *o*-xylene were 200 mg m^−3^, 150 mg m^−3^, 60 mg m^−3^, 90 mg m^−3^ and 75 mg m^−3^, respectively from day 1 to day 60, and were increased to 300 mg m^−3^, 200 mg m^−3^, 90 mg m^−3^, 120 mg m^−3^ 100 mg m^−3^ for the remaining 20 days. Both BTFs were operated at ambient temperature (26–30°C) and pressure throughout the experimental process.

### Gas chromatograph analysis

Gas samples were collected using 2 L Tedlar bags after the BTF re-started 2 hours and measured immediately by a gas chromatograph (SHIMADZU, GC-2010 plus, Japan) equipped with a capillary column (HP-INNOWAX, 30 m × 0.25 μm × 0.50 mm) and a flame ionization detector. Nitrogen was used as the carrier gas. The temperatures of the injector and detector were 200°C and 250°C, respectively. The GC oven temperature was programmed as following: 35°C for the initial 3 min increasing by 12°C min^−1^ until 180°C, after which it was left to stand for 1 min.

To evaluate the performance of the two BTFs, removal efficiency (RE) and elimination capacity (EC) for each individual VOC was calculated using Eq (1):

$$RE\;(\%)=\left(C_{in}-C_{out}\right)/C_{in}\times 100$$ (1)

$$EC\;(g\;m^{-3}\;h^{-1})=(C_{in}-C_{out})\times Q/V$$ (2)

where C_in_ and C_out_ are the inlet and outlet VOC concentrations (mg m^−3^), respectively. Q is the gas flow rate (L h^−1^), V is the volume of the empty bed volume (L).

### Measurement of environmental variables

Leachate (50 mL) was collected in triplicate from the two BTFs during the renewal of nutrient solution. After centrifugation for 10 min at 7,000 g, the supernatant was collected for physiochemical measurement. NO_3_^−^, NO_2_^−^, PO_4_^3−^ and CO_3_^2−^ were measured using ion chromatography (Thermo SCIENTIFIC, DIONEX ICS-1100, USA). Total organic carbon (TOC) was measured using a TOC analyzer (SHIMADZU, Japan).

### DNA extraction and high-throughput sequencing

The pellet from the above leachate was collected for DNA extraction. Genomic DNA was extracted using the PowerSoil DNA Isolation Kit (MO BIO Laboratories, Inc.) according to the manufacturer's instructions. Universal primers 515F (5′-GTG CCA GCM GCCGCG GTA A-3′) and 909R (5′- CCC CGY CAA TTC MTT TRA GT -3′) with 12 nt unique barcodes were used to amplify the V4 and V5 hypervariable regions of the 16S rRNA gene for sequencing. 16S rRNA gene was sequenced at School of Minerals Processing and Bioengineering, Central South University. The sequence data can be found in the NCBI Sequence Read Archive (SRA; http://www.ncbi.nlm.nih.gov/Trace s/sra/), with accession number from SRR8271629 to SRR8271655.

### Analysis of biofilm formation capacity and EPS secretion

Packing materials were sampled in triplicate on days 30, 60 and 80. Biofilm formation capacity was measured using the modified method of Pang et al. (2005) [20]. In brief, four patches of packing material were washed using phosphate-buffered saline (PBS), stained for 20 min with 30 mL 0.1% crystal violet, washed in triplicate with ultrapure water and immersed in 30 mL 95% ethanol for 20 min. Biofilm formation capacity was evaluated by absorbance at 600 nm. EPS were extracted according to the method of Jia (2017) with some modification [21]. In brief, four patches of packing material were washed with PBS. Biofilm was peeled off the packing material using a vortex and ultrasonication. The sediments were collected after centrifugation for 15 min at 2,000 g. After being resuspended to their original volumes with PBS, the solution was centrifuged at 5,000 g for 15 min and the bulk solution was collected as loosely bound EPS (LB-EPS). The sediments were resuspended and treated with ultrasonication at 40 kHz for 1 min, and then centrifuged at 17,000 g for 20 min. The bulk solution was collected as tightly bound EPS (TB-EPS). LB-EPS and TB-EPS solutions were filtered through a 0.45 μm membrane. EPS protein was quantified using the method of Bradford (1976) with bovine serum albumin as a standard [22]. Polysaccharide was quantified by the phenol-sulfuric acid method using glucose as a standard [23]. All characterizations were performed in duplicate.

### Data analysis

The raw sequence reads were quality-filtered with QIIME Pipeline (v1.9.0), including: 1) demultiplex applied to individual samples based on their unique barcodes; 2) filtering of sequences < 200 bp or with an average Q-score < 25 and; 3) identification and removal of potential chimeric sequences using the Uchime algorithm. Clean reads were clustered into operational taxonomic units (OTUs) at 97% cutoff. Taxonomic assignment of representative OTU sequences was implemented according to the RDP classifier at a confidence of 70%. The OTU relative abundance table was rarefied to 4,970 reads per sample to neutralize bias introduced by various sequencing depths. Rarefaction curves were provided in supplementary materials (S1 Fig), and the Good’s coverage were more than 98% to illustrate the sequencing data was enough to reflect the majority of the bacterial diversity in all samples. Principal coordinates analysis (PCoA) was explored based on Bray-Curtis distance using Past (v3.0) to display the shift in the structure of microbial community between the two BTFs. Canonical correspondence analysis (CCA) was performed using R (vegan package) to reveal which environmental parameter(s) contributed to the divergence of microbial communities between the two BTFs; only the OTUs with relative abundance > 0.1% were included. The linear discriminant analysis (LDA) effect size (LEfSe) method (http://huttenhower.sph.harvard.edu/lefse/, last accessed 2 December 2018) was used to detect potential biomarkers in BTF1 and BTF2 based on normalized relative abundance.

## Results

### Biological removal of the intermittent VOCs in the biotrickling filter

**Fig 1:**
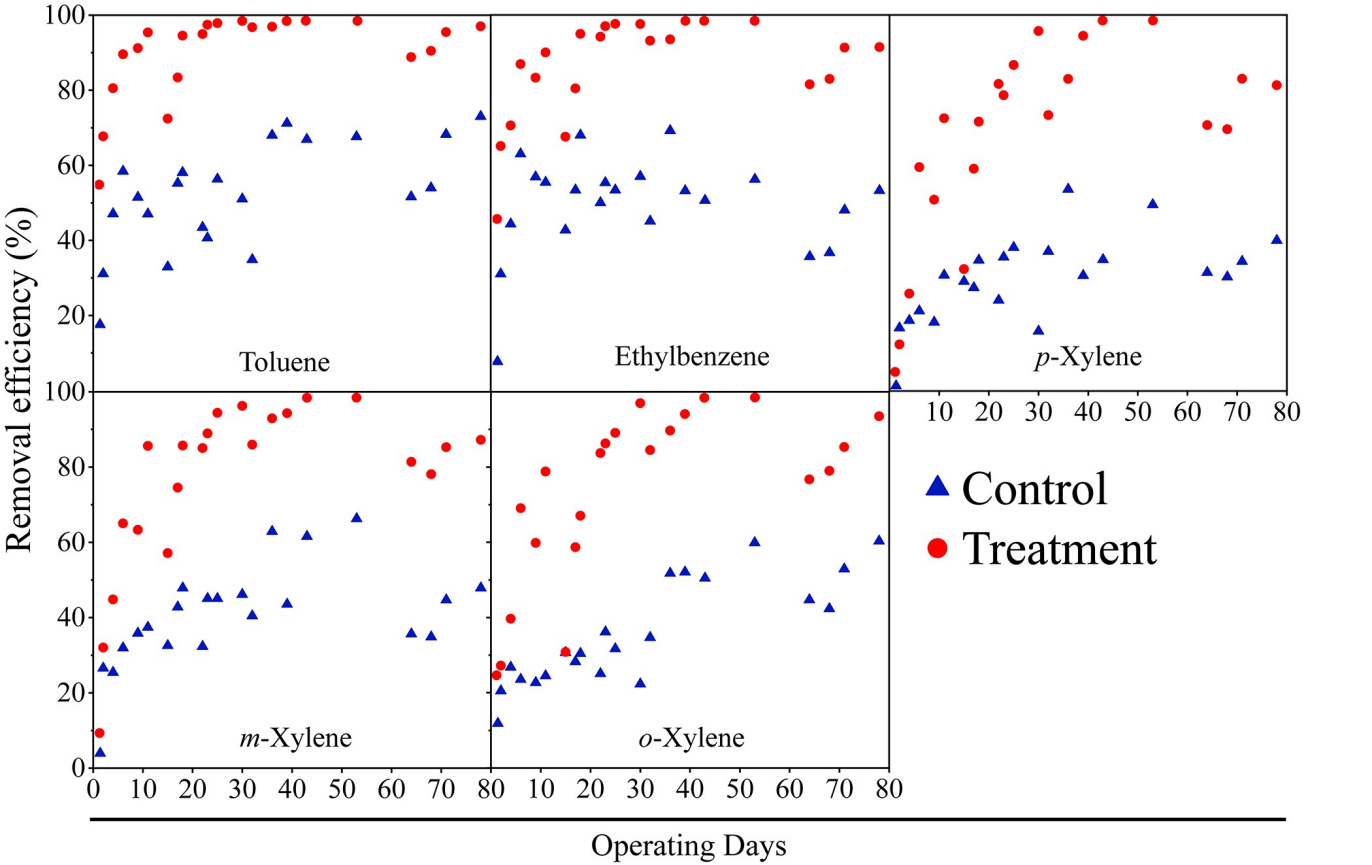
Removal efficiency of the five aromatic compounds in the two BTFs.

To verify the industrial application of the modified packing materials, removal performances were monitored along 80 days. Fig 1 demonstrates the removal performances of the two BTFs on the five aromatic compounds. BTF2 outperformed BTF1 from the start of treatment and remained superior throughout the experiment. By day 11, the BTF2 removal efficiencies for toluene, ethylbenzene, *p*-xylene, *m*-xylene and *o*-xylene were 95.36%, 90.02%, 72.51%, 85.58% and 78.80%, respectively, whereas those of BTF1 were only 47.01%, 55.44%, 30.80%, 37.41% and 24.48%, respectively. After the renewal of nutrient solution on day 11, sharp decreases in removal performance of both BTFs were found over the following few days, indicating that biofilm formation was incomplete and a large portion of functioning microorganisms were in planktonic status. Toluene was among the most easily removed pollutants in BTF2, followed by ethylbenzene, with their removal efficiencies exceeding 94% from day 18. The three xylene isomers were removed less easily during the first month, with their removal efficiencies only reaching 94% by day 39. However, nearly complete removal of all pollutants was achieved on day 43. In comparison, the removal efficiency of BTF1 never exceeded 72% within the first 60 days. After inlet concentrations were increased by 1.5-fold on day 61, significant drops in removal efficiency were detected for both BTFs; however, BTF2 was less affected. The performances of the two BTFs were also assessed in terms of their elimination capacities (ECs). The maximum EC value observed in BTF1 was 30.43 g m^-3^ h^-1^, which was achieved at inlet loading rates of 180 L h^-1^. By comparison, BTF2 achieved maximum EC of 47.56 g m^-3^ h^-1^ at the same inlet loading rates.

### Determination of physiochemical indexes in leachate and canonical correspondence analysis

The present study modified packing materials with sodium alginate and polyvinyl alcohol. Daily monitoring of concentrations of NO_3_^−^, NO_2_^−^, PO_4_^3−^, CO_3_^2−^ and leachate TOC was implemented for 31 consecutive days (S1 Table). The results showed that the two BTFs differed considerably in TOC concentration, except when nutrient solutions were renewed on day 0 and day 12. TOC concentration of the BTF2 nutrient solution increased drastically from 91.0 mg L^−1^ to 240.2 mg L^−1^ only one day after the startup of the reactor, and was maintained at approximately 300 mg L^−1^ during the first nutrient cycle. The peak TOC concentration detected in BTF2 was 414.0 mg L^−1^, 40-fold higher than that in BTF1. In contrast, TOC concentration in BTF1 decreased significantly with running time during the first nutrient cycle, and ranged between 7.30 mg L^−1^ and 91.0 mg L^−1^. The result verified that the modifiers gradually dissolved in the leachate, and thus could act as a soluble carbon source to support the growth of microorganisms during nutrient-poor conditions.

TOC concentrations in the solutions of both BTFs greatly decreased after renewal of nutrient solutions on day 12. However, the overall concentration of TOC in BTF2 remained significantly higher than that in BTF1, suggesting that even during the second nutrient cycle, a large amount of the modifier was being released from the packing bed in BTF2. Besides, the concentrations of nitrate nitrogen were significantly less in BTF2 than in BTF1, demonstrating that more nitrate nitrogen was consumed in BTF2. The quantitative determination also showed that more carbonate was accumulated inside BTF2. Together with results confirmed that microorganisms inside BTF2 undergone a rapid proliferation and showed more activities in the biotransformation of VOCs.

**Fig 2:**
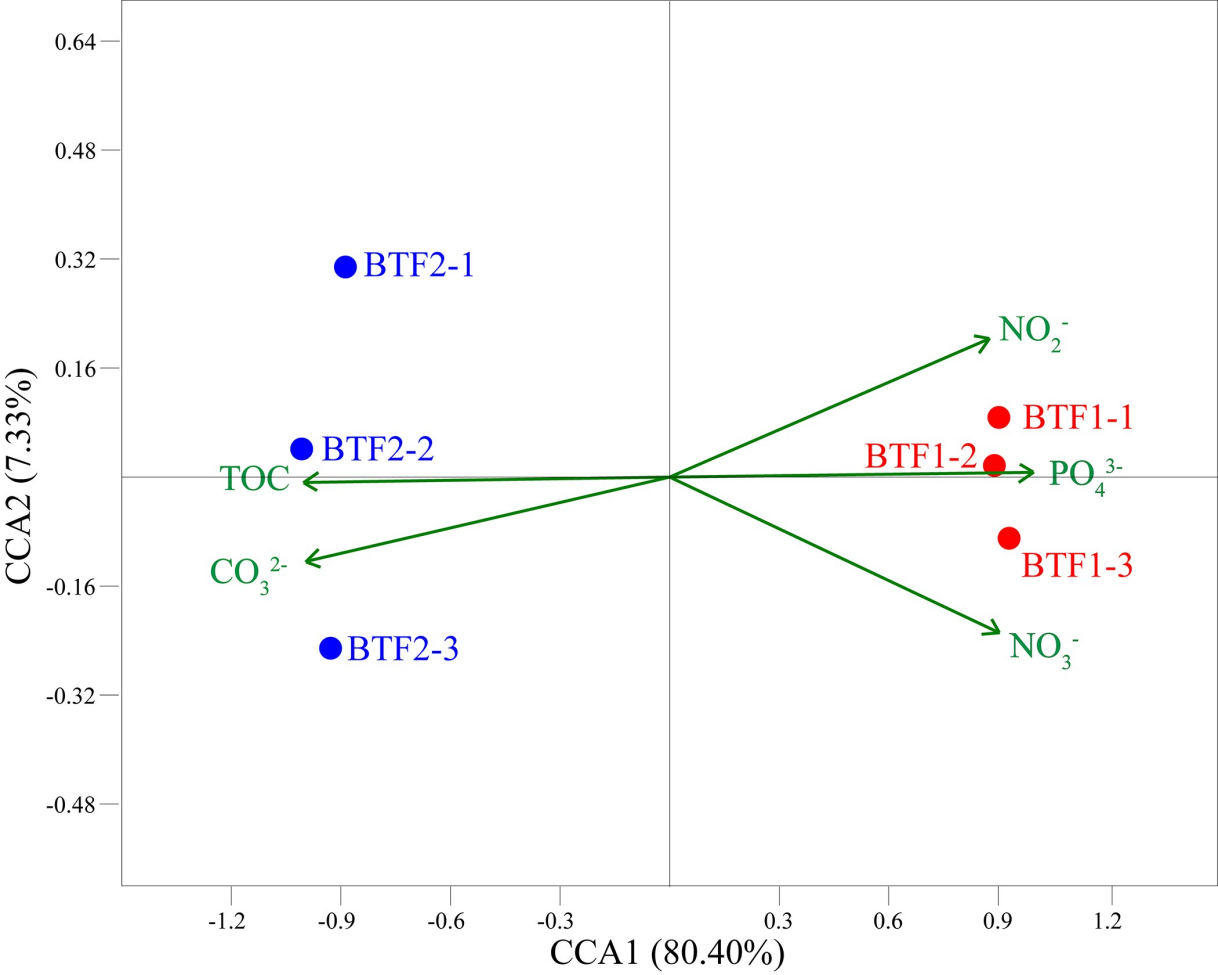
Species-parameter biplot displaying samples from the two BTFs on day 11 with respect to environmental parameters based on CCA.

The relationships between environmental parameters and microbial communities were revealed by CCA (Fig 2). NO_3_^−^, NO_2_^−^, PO_4_^3−^, CO_3_^2−^ and TOC with variance inflation factors (VIF) < 20 explained 92.93% of the total variance of microbial communities. The first two axes of CCA explained 87.73% of the variance. Samples from the two BTFs collected on day 11 were greatly distant from each other. Microbial communities from BTF2 were distributed along higher TOC and CO_3_^2−^ gradients. Mantel test also proved that TOC had a highly positive correlation with microbial communities (r^2^ = 0.9997, *p* = 0.001). Altogether, the modified packing material not only promoted biofilm formation by providing an additional carbon source, but also played critical roles in shaping microbial communities.

### Identification of microbial biomarkers during the early acclimation stage

**Fig 3:**
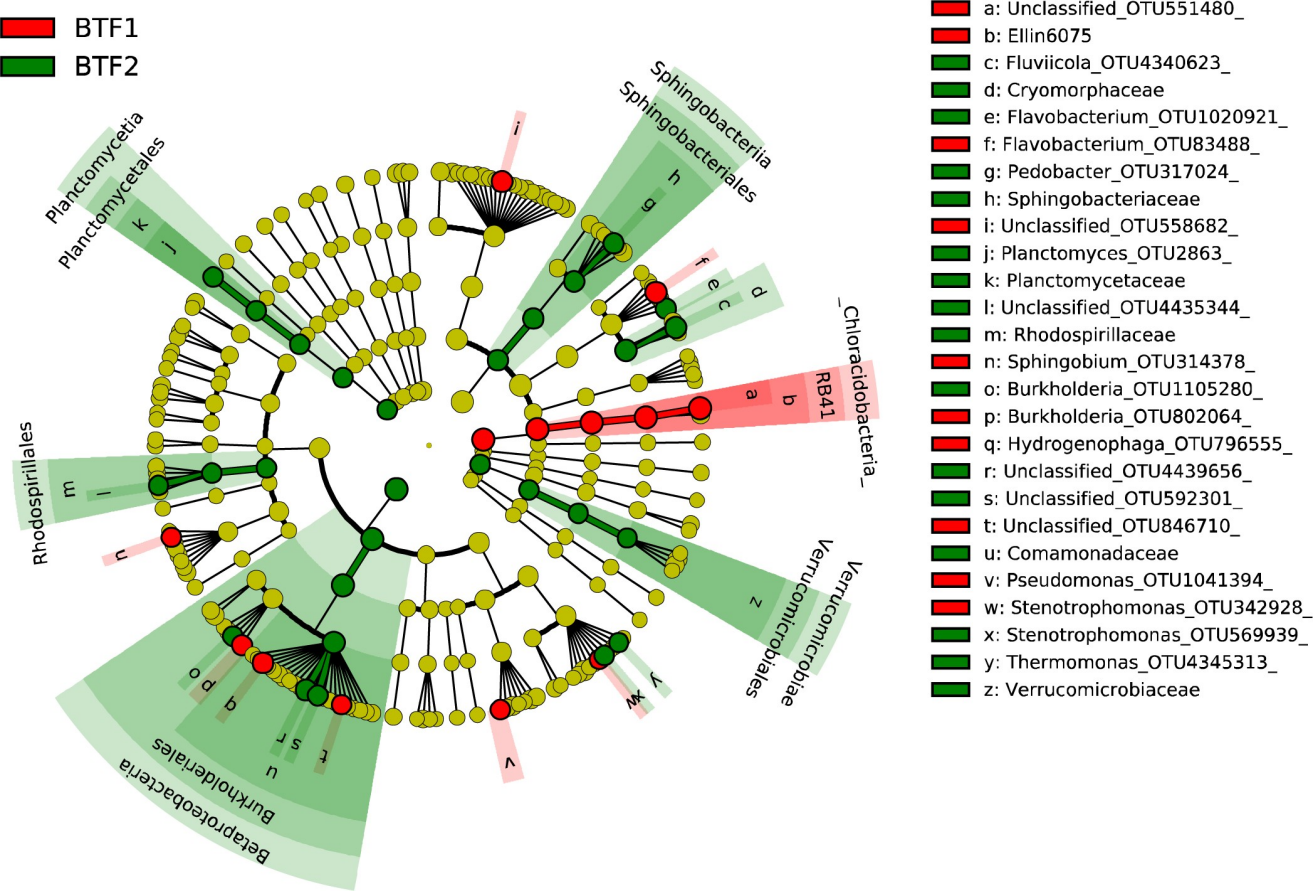
Linear discriminant analysis (LDA) effect size taxonomic cladogram highlighting the microbial biomarkers that statistically and biologically differentiate between the two BTFs. Significant (red and green) and non-significant (yellow) discriminant taxonomic nodes are colored. Circle diameter is proportional to the taxon's abundance.

Microbial communities were analyzed to identify which microbes were specifically adhered in BTF2. Six samples were collected on day 11 from both BTFs. A total of 594 OTUs were obtained by high-throughput sequencing of 16S rRNA genes. Of these, 155 had a relative abundance > 0.1%, and accounted for 97.5% and 95.8% of all identified OTUs in BTF1 and BTF2, respectively. To identify the microbial biomarkers with statistically significant differences between BTF1 and BTF2, we performed linear discriminant effect size analysis (LEfSe) based on the samples collected on day 11 (Fig 3). LEfSe detected 19 differential OTUs with LDA scores > 4.0 (S2 Fig). Of special note is that all microbial biomarkers were also the predominant bacterial taxa in either BTF1 or BTF2.

These biomarkers were assigned to the four major bacterial phyla including Acidobacteria, Bacteroidetes, Proteobacteria and Planctomycetes. Phylum Acidobacteria was overrepresented in BTF1 with an average relative abundance of 29.23%, far greater than that in BTF2 (0.17%). In contrast, Phylum Planctomycetes predominated in BTF2 (3.00%), whereas it was hardly occurred in BTF1 (0.01%). Enriched members of Bacteroidetes and Proteobacteria were found in both BTFs, whereas they differed considerably in the distribution of sub-taxonomic units. At the genus level, BTF1 harbored abundant bacteria in the genera *Flavobacterium* (13.76%), *Hydrogenophaga* (7.12%), *Burkholderia* (4.36%), *Pseudomonas* (2.70%), *Sphingobium* (2.63%), *Stenotrophomonas* (2.13%) and OTU551480 (of phylum Acidobacteria, 29.24%), OTU558682 (of family Chitinophagaceae, 11.86%) and OTU846710 (of family Comamonadaceae, 2.60%). OTU4439656 (of family Comamonadaceae, 12.80%) was the most predominant species in BTF2, followed by genera *Fluviicola* (12.09%), *Flavobacterium* (5.56%), *Pedobacter* (4.45%), OTU592301 (of family Comamonadaceae, 4.37%), *Burkholderia* (3.32%), *Planctomyces* (3.00%), *Stenotrophomonas* (2.78%), OTU4435344 (of family Rhodospirillaceae, 2.36%) and *Thermomonas* (2.09%).

### Compositions of EPS and the microbial community after depletion of the modifiers

The concentration of TOC in BTF2 greatly decreased to a very low level from day 25, without showing significant difference with that of BTF1, indicating that modifiers on the surface of packing material in BTF2 had been depleted completely. Results showed that the capacity of BTF2 to form biofilm was approximately four-fold higher than that observed in BTF1 (Table 1). The quantity of EPS secreted in BTF2 on day 60 was ten-fold higher than in BTF1 (Table 1). So, it can be concluded that using modified packing materials undoubtedly speeded up the biofilm formation. Biofilm comprised largely of protein, and to less extent, polysaccharide in both BTFs. BTF2 showed a higher ratio of protein to polysaccharide during the first 60 days compared to BTF1. What is more interesting is that the removal efficiencies of the five pollutants significantly correlated with the protein content, with coefficients ranged from 0.890 to 0.942 (S2 Table). In comparison, the contents of polysaccharide did not show any significant correlation with removal efficiency of all pollutants. A sharp reduction in biofilm formation capacity was observed in both BTFs once higher VOC loading was implemented on day 80. However, the extent of reduction was lower in BTF2, with a reduction of 51.36% and 41.89% in the bottom layers of BTF1 and BTF2, respectively.

**Table 1:** Biofilm formation capacity and EPS production during the operation time.

		BFC	EPS-protein (ug/ cm^3^_PU)	EPS-polysaccharide (ug/ cm^3^_PU)
**BTF1**	30-U	2.80±0.23	0.00±0.00	1.70±0.09
	30-B	3.94±0.31	1.89±0.33	1.46±0.05
	60-U	6.36±0.86	0.33±0.13	0.91±0.15
	60-B	7.73±1.48	5.42±0.66	1.85±0.30
	80-U	3.20±0.52	4.77±0.33	0.35±0.09
	80-B	3.76±0.30	12.42±2.20	2.03±0.36
**BTF2**	30-U	9.96±0.30	6.65±0.54	3.74±0.10
	30-B	14.30±0.44	32.35±1.15	4.11±0.20
	60-U	24.84±5.36	11.12±3.75	4.86±0.27
	60-B	31.08±4.10	49.16±3.11	6.14±0.15
	80-U	11.97±1.23	17.04±0.66	3.45±0.35
	80-B	18.06±1.25	43.97±3.43	10.60±0.20

Note: 30, 60, and 80 were the operating days when the samples were collected and measured; U presented the upper layer in a BTF, and B presented the bottom layer in a BTF; BFC represents biofilm formation capacity.

Microbial diversity (as indicated by Shannon_H) and microbial evenness (as indicated by Simpson_1-D) in BTF2 were consistently higher than that in BTF1, with the differences varying considerably over the operating time (S3 Table). Pearson and Spearman correlations showed that both the Shannon_H and Simpson_1-D indices were positively correlated with the removal efficiencies of the five pollutants (r > 0.80 and *p* < 0.05), partially explaining the different removal performances between the two BTFs. Microbial diversity and evenness decreased in both BTFs when inlet loading increased. However, BTF2 maintained relatively higher levels over the entire operation time.

**Fig 4:**
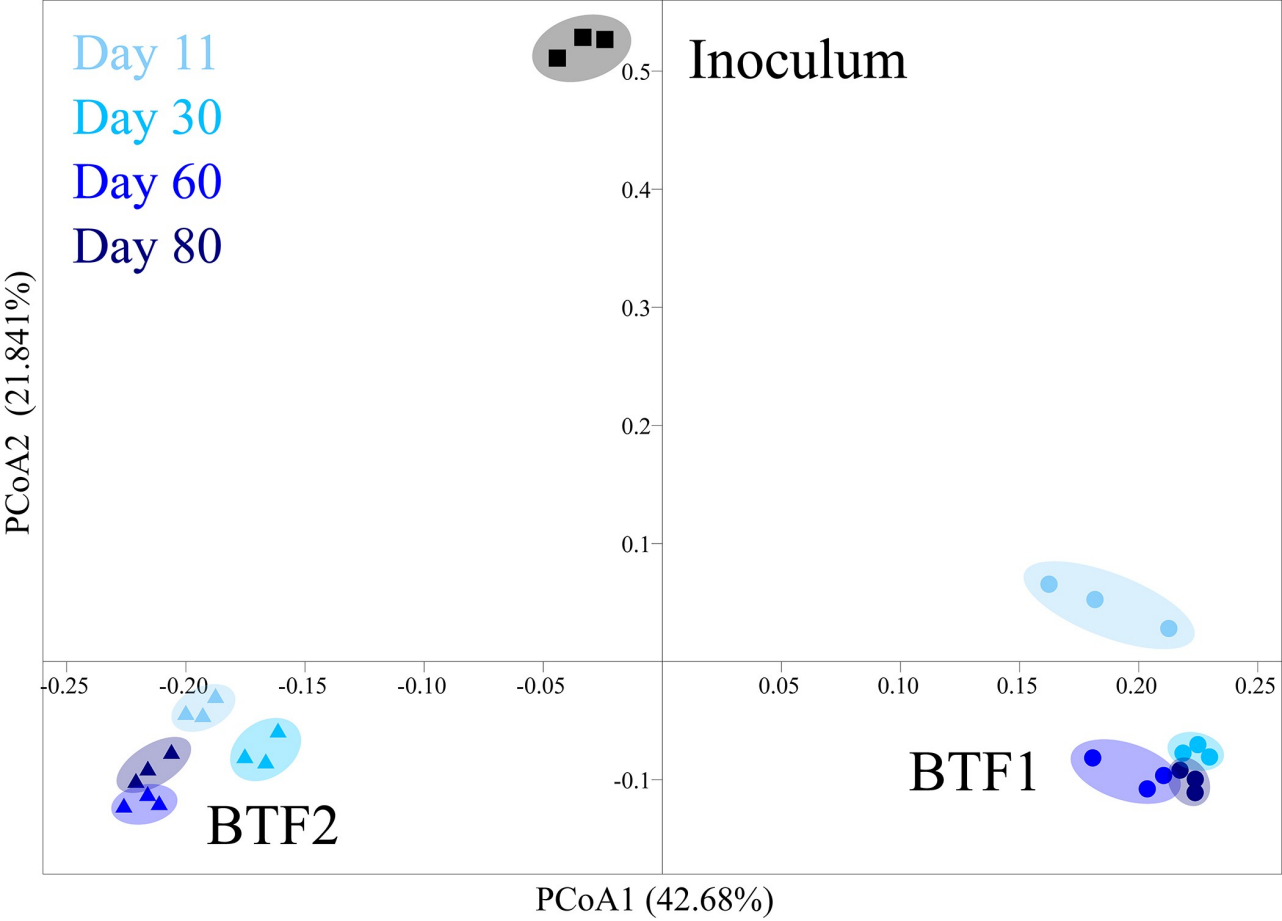
Weighted principal coordinate analysis (PCoA) of microbial communities. Sample from initial inoculums are showed with squares. Sample from BTF1 are showed with circles. Sample from BTF2 are showed with triangles.

To further understand the dynamics of the microbial communities in the two BTFs after depletion of the modifiers, microbial community structures were analyzed using the 16S rRNA gene high-throughput sequencing method. The numbers of OTUs were 94–286 per sample. Weighted principal coordinate analysis (PCoA) was performed using the OTU data matrix based on the Bray-Curtis distance (Fig 4) to illustrate the differences in microbial communities between the two BTFs over the course of the treatment. A total of 64.52% of the variance in data could be explained by the first two principal coordinate axes. PCoA showed that microbial communities in both BTFs shifted away from that of the seed community. BTF1 and BTF2 were clustered into two distinctly different groups along PCoA 1. Stability of the microbial community was maintained over the entire operating period for both BTF1 and BTF2, even when inlet loading was increased. The results showed that both microbiome membership and abundance were much more different between BTFs than between operating stages.

**Fig 5:**
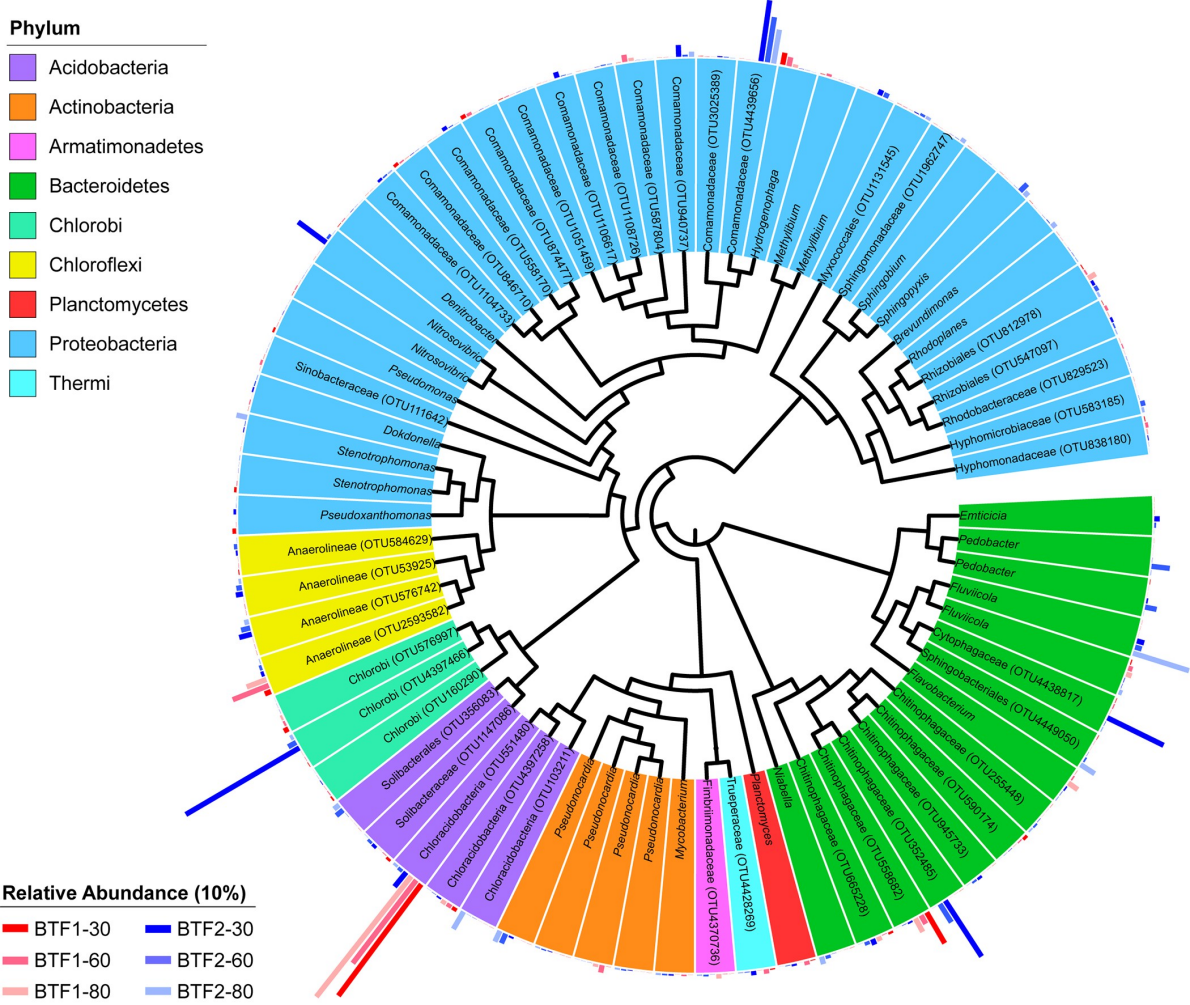
The dendrogram of the most abundant 69 species on day 30, 60 and 80 in the two BTFs.

To illustrate the microbial compositions of BTF1 and BTF2 in detail, relative abundances at the phylum level was showed in S4 Table, and 69 OTUs with higher relative abundance were drawn as a dendrogram (Fig 5). In Fig 5, different phyla were indicated by different colors, and the histograms exhibit relative abundances of corresponding OTUs, which illustrates that the dominant microbes differed between the two BTFs. OTU551480 of class Chloracidobacteria was the most enriched species in BTF1, with 39.73% relative abundance. OTU4439656 (of family Comamonadaceae, 17.29%) and genus *Pedobacter* were the two most-abundant species in BTF2. It is worth noting that these OTUs also were the microbial biomarkers in their respective BTF on day 11. It was illustrated that the predominant microbial biomarkers that were enriched during the early acclimatization stage subsequently went on to define the microbial compositions during the biofilm formation and maturation process. The unique microbial biomarkers colonized in BTF2 effected by sodium alginate generated a distinct microbial community over the operating time, and facilitated higher pollution degradation capacity and biofilm formation.

## Discussion

The biological removal of mixed gases containing intermittent VOCs by biofiltration presents a major challenge [24], and improvement to existing methods have been rarely reported. Li and Moe (2005) tested the effectiveness of a hybrid biofilter column combined with a granular activated carbon (GAC) column and found that the GAC column efficiently buffered the peak inlet loading of mixed acetone and toluene, thereby sharply minimized the negative effect resulting from carbon starvation during the idle phase [25]. However, the aforementioned study only tested two components of VOCs, and VOCs removal efficiency was unreliable due to the selectivity of GAC on different components [26]. The present study aimed to simultaneously rapidly acclimate microorganisms and remove mixed VOCs under intermittent VOCs loading using a single biofilter column packed with modified polyurethane foam. Even though the modifiers were completely depleted after some time, biofilm had successfully formed and matured before that (Table 1). The mature biofilm formed within BTF2 is the leading cause of its superior removal performance throughout whole experimental time even when the VOCs inlet increased by 1.5-fold (Fig 1). The results of the present study show that polyurethane foam modified with a mixture of 3% sodium alginate and 5% polyvinyl alcohol presents a promising packing material for use in a bioreactor to remove intermittent VOCs in industrial application.

As noted by Goldford et al. (2018), community-level functions were highly governed by nutrient availability. Under an intermittent mode, the loading of VOCs was paused over nights and weekends [27]. The lack of a carbon source resulted in the degradation of microorganism intracellular carbon storage, the changes of metabolic pathways, formation of spores or other resistant structures, or simply death [28]. The use of sodium alginate and polyvinyl alcohol in the present study managed to bypass nutrient starvation by providing an alternative form of organic carbon during the early acclimation stage (S1 Table), thereby significantly enhancing biofilm formation and maturation, and then improving the removal performance of BTFs. (Table 1 and Fig 1).

On one hand, the addition of organic carbon at startup stimulated EPS secretion (Table 1). It was reported that EPS promoted cohesion between cells and of cells to surfaces, thereby contributing to biofilm formation and maturation [12, 15]. In addition, EPS provided structural support and played an important role in maintaining 3-D structures of biofilm [29]. The addition of sticky sodium alginate and polyvinyl alcohol was beneficial to EPS secretion as it irreversibly raised the opportunity for cell adhesion, which was a key step within biofilm formation and maturation. Once matured, biofilm persisted and could provide a physical and structural barrier against external stimuli [12]. The biofilm formed in BTF2 remained more mature, with more EPS and higher biofilm formation capacity, even when modifiers were completely depleted and the inlet loading increased (Table 1 and S3 Table). In addition, it has been reported that EPS was responsible for the adsorption and mass transfer of contaminants [30, 31]. Mass transfer has been reported as a critical rate-limiting factor during biofiltration of many VOCs [32–34]. Thus, EPS played an important role in the degradation of VOCs. Higher EPS secretion for the most part could explain higher removal efficiencies of BTF2. Proteins and polysaccharides are the two main components of EPS [35], and it is generally considered that the proteins constitute the hydrophobic fraction of EPS whereas the polysaccharides are hydrophilic compounds. The five pollutants used in the present study could be categorized as moderately hydrophobic substances, and the hydrophobic fraction of EPS was more beneficial for their absorption. Our results showed that the protein portion in EPS was positively correlated with the removal efficiency of toluene, ethylbenzene, *p*-xylene, *m*-xylene and *o*-xylene, whereas the polysaccharide portion was not (S2 Table). The positive correlation between protein content and the removal efficiencies of the five pollutants suggested that the hydrophobicity of protein may help promote the mass transfer of these hydrophobic VOCs. Furthermore, studies reported that fractional EPS could serve as a source of carbon or energy under nutrient-poor conditions for the growth of diverse microorganisms [14].

On the other hand, the addition of organic carbon at startup allowed the unique biomarkers or pioneering microorganisms to colonize within the BTF. Species that were slow growing but resilient to nutrient poor conditions tended to dominate the control BTF (BTF1), whereas fast-growing species able to utilize polysaccharides tended to dominate the treatment BTF (BTF2). It showed that OTUs of the phylum Acidobacteria and family Chitinophagaceae were dominant biomarkers in BTF1 (Fig 3). Members of phylum Acidobacteria were reported to be able to degrade and utilize a diverse range of structural and storage polysaccharides to increase resilience under nutrient-limited conditions, and therefore played an important role in ecological niches, particularly in the absence of fast‐acting degraders [36]. A dormant stage called a microcyst was reported in some members of family Chitinophagaceae to resist adverse conditions [37]. These microbial cells should be characterized by higher substrate uptake rates, particularly during times of inactivity, to facilitate higher probabilities of dominance during nutrient-limited conditions [38]. In contrast, BTF2 harbored unique biomarkers, such as members of phylum Planctomycetes and genus *Flavobacterium* (Fig 3). All described species within Planctomycetes are characterized by the ability to grow on sugar monomers, disaccharides and polysaccharides [39]. Most *Flavobacterium* species are able to degrade a variety of polysaccharidic components of algae, agar, alginate, chitin and laminarin. Some species can survive and grow in nutrient-poor conditions by degrading intracellular polymers or the use of a dormant stage, whereas other species grow in nutrient-rich conditions by degrading polysaccharides, such as sodium alginate.

There have been few studies to determine whether pioneer colonizers could maintain their predominance during the whole operating time in BTFs for treating VOCs, although some analogues were reported in water distribution system. Doğruöz et al. (2010) was not able to identify pioneer colonizer microorganisms in biofilms after 216 h–456 h in a simulated recirculating cooling-water system [40]. Douterelo et al. (2014) found that after initial colonization by a few bacterial species in a chlorinated experimental drinking water distribution system, they lost their dominance after 28 d [41]. Szewzyk et al. (2000) proposed that secondary colonizers were able to attach to growing biofilm in a drinking-water distribution system [42]. In the current study, it showed that the predominant biomarkers or pioneering microorganisms acclimatized in the two BTFs maintained their high relative abundances during the biofilm formation process (Fig 5). Therefore, the biomarkers were to a large extent responsible for the development of microbial community structures. The presence of higher biodiversity and evenness within the microbial communities in BTF2 resulted from the stimulation of the unique biomarkers by the attached modified polyurethane foams (S4 Table). It was reported that greater diversity is associated with higher ecosystem stability [43, 44]. Wittebolle et al. (2009) demonstrated communities with higher evenness exhibited stronger functional robustness [45]. Thus, BTF2 was able to maintain stability and resilience, even when modifiers were subsequently depleted completely or VOC inlet loading was increased, to explain for the improved removal performance over the entire operating time.

## Conclusions

The present study mainly considered microbial biomarkers that were adhered on surfaces and the key environmental factors responsible. However, irreversible adhesion of cells onto surfaces is a key step during the formation and maturation of biofilm, and is affected by many factors including physical and chemical properties of the surfaces [15]. Physicochemical properties of packing materials, such as hydrophobicity, surface roughness and topography, should be taken into consideration in future research. In addition, biofilm formation is regulated by complex networks of genes, and quantitative polymerase chain reaction (qPCR) of some functional genes may be explored to illuminate possible underlying mechanisms.

## Supporting information

S1 Fig The rarefaction curve for observed OTUs. (TIF)

S2 Fig Histogram of differentially abundant features between the two BTFs (logarithmic LDA score>4.0 and p<0.05). (TIF)

S1 Table Concentrations of NO_3^-^_, NO_2^-^_, PO_4_^3_-_^, CO_3_^2_-_^ and TOC in nutrient solution during the first 31 days. (DOCX)

S2 Table Pearson’ linear correlations between biofilm formation capacity and EPS with removal efficiency of the five pollutants. (DOCX)

S3 Table Microbial α-diversity metrics at different BTFs and time. (DOCX)

S4 Table Relative abundances of dominant phyla during the operating time in the two BTFs. (DOCX)
