# Supplementary figures and images for: Enhanced biological removal of intermittent VOCs and deciphering the roles of sodium alginate and polyvinyl alcohol in biofilm formation
Source: PLoS One. 2019 May 22;14(5):e0217401. doi: 10.1371/journal.pone.0217401 (PMC6530866; doi:10.1371/journal.pone.0217401)

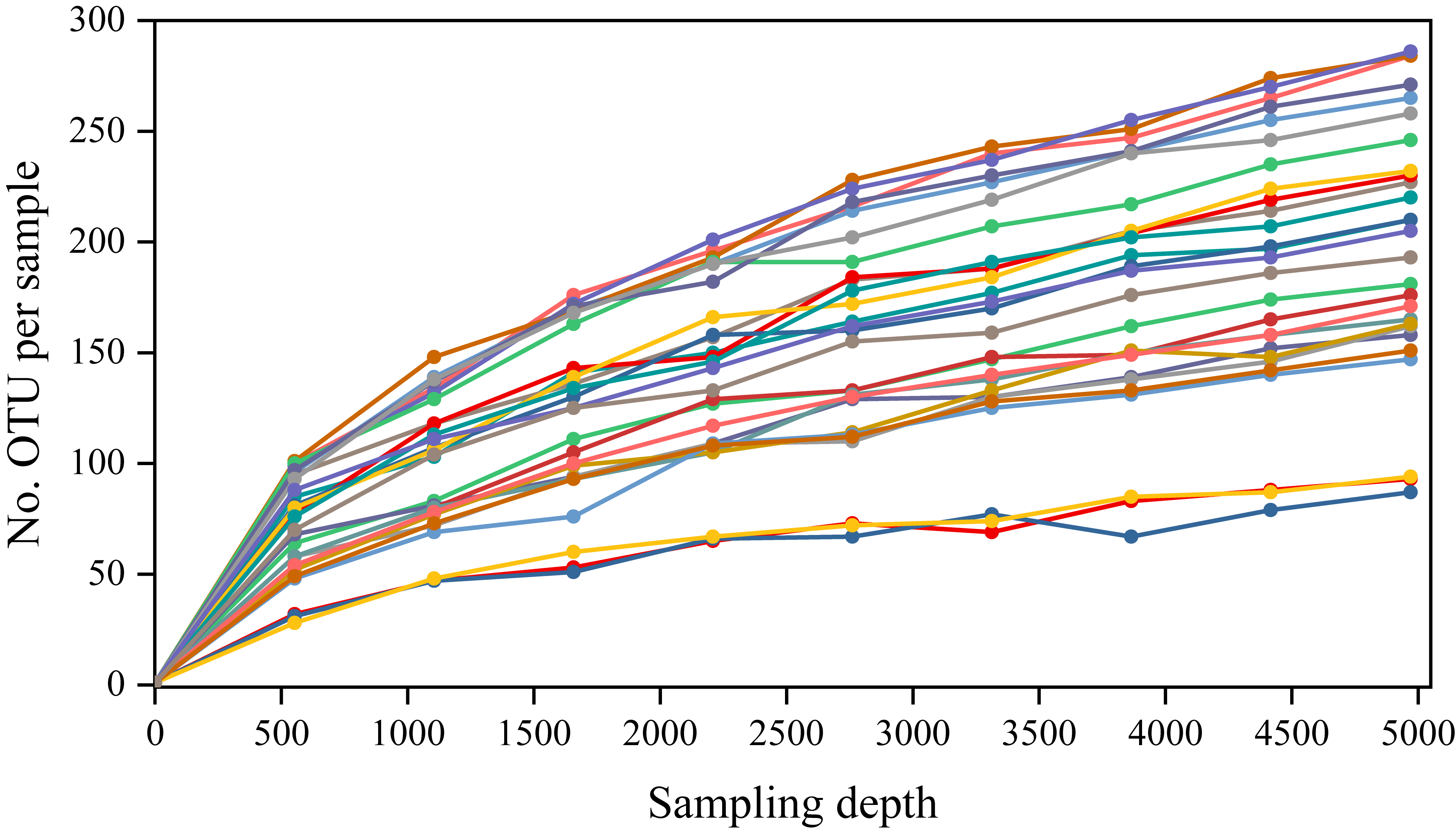

Supplement: S1 Fig — (TIF) [file pone.0217401.s001.tif]

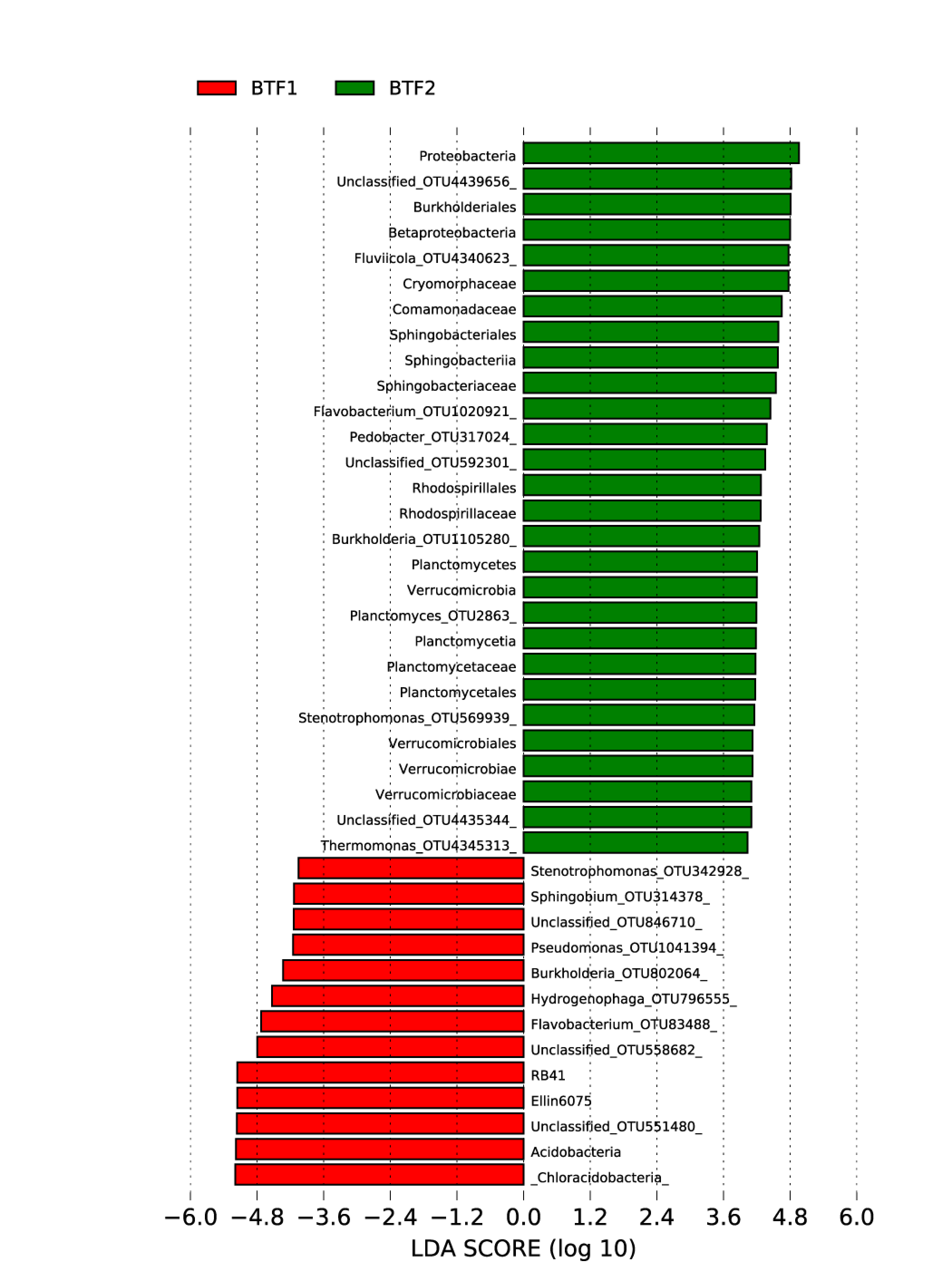

Supplement: S2 Fig — (TIF) [file pone.0217401.s002.tif]
