# Supplementary material for: Enhanced biological removal of intermittent VOCs and deciphering the roles of sodium alginate and polyvinyl alcohol in biofilm formation
Source: PLoS One. 2019 May 22;14(5):e0217401. doi: 10.1371/journal.pone.0217401 (PMC6530866; doi:10.1371/journal.pone.0217401)
Supplement: S1 Table — (DOCX) [file pone.0217401.s003.docx]

S1 Table. Concentrations of NO_3_^-^, NO_2_^-^, PO_4_^3-^, CO_3_^2-^ and TOC in nutrient solution during the first 31 days.

| time | NO_3_^-^ (mM) | | NO_2_^-^ (mM) | | PO_4_^3-^ (mM) | | CO_3_^2-^ (mM) | | TOC（mg/L） | |
| --- | --- | --- | --- | --- | --- | --- | --- | --- | --- | --- |
| day | BTF1 | BTF2 | BTF1 | BTF2 | BTF1 | BTF2 | BTF1 | BTF2 | BTF1 | BTF2 |
| 0 | 36.83 | 36.83 | 0.00 | 0.00 | 26.64 | 26.64 | 35.45 | 35.45 | 90.96 | 90.96 |
| 1 | 29.43 | 20.73 | 0.00 | 0.00 | 21.00 | 13.28 | 40.45 | 41.09 | 60.14 | 240.20 |
| 2 | 28.99 | 23.79 | 0.00 | 0.00 | 20.39 | 7.86 | 39.58 | 44.76 | 26.52 | 353.40 |
| 3 | 29.91 | 23.02 | 0.00 | 0.00 | 22.51 | 6.24 | 46.28 | 51.05 | 10.35 | 414.00 |
| 4 | 31.06 | 21.55 | 0.00 | 0.00 | 22.02 | 6.07 | 45.93 | 55.01 | 8.80 | 373.00 |
| 5 | 32.96 | 23.10 | 0.00 | 0.00 | 23.59 | 6.09 | 45.29 | 65.32 | 7.27 | 317.90 |
| 6 | 29.32 | 23.02 | 0.58 | 0.00 | 20.40 | 5.68 | 43.95 | 64.50 | 12.56 | 286.00 |
| 7 | 28.80 | 23.32 | 0.72 | 0.00 | 20.53 | 5.92 | 40.69 | 73.35 | 9.33 | 284.10 |
| 8 | 27.93 | 23.80 | 0.48 | 0.00 | 18.71 | 5.70 | 35.27 | 74.87 | 12.98 | 295.70 |
| 9 | 28.83 | 23.11 | 0.59 | 0.00 | 21.36 | 5.81 | 40.63 | 80.11 | 12.22 | 319.70 |
| 10 | 27.87 | 23.60 | 0.58 | 0.36 | 20.32 | 5.97 | 29.33 | 80.46 | 11.35 | 321.20 |
| 11 | 29.33 | 24.52 | 0.85 | 0.40 | 23.76 | 5.87 | 34.46 | 91.11 | 14.65 | 324.20 |
| 12 | 42.95 | 42.95 | 0.00 | 0.00 | 46.78 | 46.78 | 6.27 | 6.27 | 21.70 | 21.70 |
| 13 | 37.17 | 35.16 | 0.00 | 0.18 | 41.05 | 26.26 | 3.01 | 44.59 | 8.09 | 134.20 |
| 14 | 37.68 | 32.55 | 0.00 | 0.26 | 39.90 | 23.31 | 7.21 | 45.35 | 9.34 | 157.80 |
| 15 | 38.02 | 32.78 | 0.00 | 0.25 | 41.50 | 19.68 | 5.40 | 52.80 | 9.44 | 85.74 |
| 16 | 38.38 | 31.01 | 0.00 | 0.17 | 39.24 | 20.50 | 9.59 | 56.53 | 8.75 | 70.52 |
| 17 | 36.32 | 32.48 | 0.00 | 0.28 | 32.24 | 17.51 | 3.01 | 57.52 | 8.67 | 67.23 |
| 18 | 36.26 | 32.84 | 0.00 | 0.19 | 38.99 | 21.49 | 6.97 | 57.86 | 7.93 | 58.40 |
| 19 | 36.77 | 32.84 | 0.00 | 0.35 | 34.95 | 21.18 | 5.58 | 59.79 | 9.97 | 53.09 |
| 20 | 35.21 | 31.34 | 0.00 | 0.48 | 37.17 | 20.46 | 5.81 | 54.66 | 7.98 | 49.77 |
| 21 | 34.21 | 30.79 | 0.00 | 0.42 | 33.42 | 19.65 | 7.90 | 57.34 | 10.34 | 53.33 |
| 22 | 32.80 | 30.30 | 0.00 | 0.14 | 35.06 | 16.30 | 11.22 | 50.00 | 10.73 | 41.61 |
| 23 | 31.92 | 29.37 | 0.00 | 0.28 | 34.65 | 18.19 | 15.36 | 62.99 | 20.93 | 44.50 |
| 24 | 31.32 | 31.24 | 0.00 | 0.10 | 35.51 | 17.77 | 12.10 | 69.69 | 20.25 | 40.16 |
| 28 | 45.72 | 43.06 | 0.00 | 0.00 | 41.45 | 38.82 | 16.17 | 22.00 | 14.80 | 17.06 |
| 29 | 43.63 | 35.96 | 0.00 | 0.00 | 39.72 | 30.58 | 16.87 | 37.19 | 14.02 | 18.13 |
| 30 | 44.32 | 34.66 | 0.00 | 0.00 | 43.36 | 34.02 | 17.45 | 50.64 | 15.42 | 16.13 |
| 31 | 43.40 | 34.37 | 0.00 | 0.00 | 42.24 | 30.94 | 18.44 | 58.10 | 18.05 | 22.90 |
