# Supplementary material for: Enhanced biological removal of intermittent VOCs and deciphering the roles of sodium alginate and polyvinyl alcohol in biofilm formation
Source: PLoS One. 2019 May 22;14(5):e0217401. doi: 10.1371/journal.pone.0217401 (PMC6530866; doi:10.1371/journal.pone.0217401)
Supplement: S2 Table — (DOCX) [file pone.0217401.s004.docx]

S2 Table. Pearson’ linear correlations between biofilm formation capacity and EPS with removal efficiency of the five pollutants.

|  | Biofilm formation capacity | protein | Polysaccharide | LB- polysacc-haride | TB- polysacc-haride | LB- protein | TB- protein |
| --- | --- | --- | --- | --- | --- | --- | --- |
| Toluene | 0.810 | 0.942^**^ | 0.747 | 0.907^*^ | 0.565 | 0.945^**^ | 0.930^**^ |
| ethylbenzene | 0.868^*^ | 0.933^**^ | 0.731 | 0.908^*^ | 0.541 | 0.950^**^ | 0.905^*^ |
| *p*-xylene | 0.855^*^ | 0.910^*^ | 0.664 | 0.866^*^ | 0.468 | 0.928^**^ | 0.882^*^ |
| *m*-xylene | 0.877^*^ | 0.890^*^ | 0.673 | 0.847^*^ | 0.492 | 0.912^*^ | 0.855^*^ |
| *o*-xylene | 0.797 | 0.906^*^ | 0.707 | 0.863^*^ | 0.532 | 0.911^*^ | 0.893^*^ |
