# Supplementary material for: Enhanced biological removal of intermittent VOCs and deciphering the roles of sodium alginate and polyvinyl alcohol in biofilm formation
Source: PLoS One. 2019 May 22;14(5):e0217401. doi: 10.1371/journal.pone.0217401 (PMC6530866; doi:10.1371/journal.pone.0217401)
Supplement: S3 Table — (DOCX) [file pone.0217401.s005.docx]

S3 Table. Microbial α-diversity metrics at different BTFs and time

|  | time | diversity | | evenness |
| --- | --- | --- | --- | --- |
|  |  | Shannon_H | Chao-1 | Simpson_1-D |
| Inoculum | 0 | 2.03±0.10 | 159.17±9.17 | 0.79±0.02 |
| BTF1 | 30 | 2.26±0.21 | 333.23±9.26 | 0.70±0.05 |
|  | 60 | 2.85±0.11 | 327.83±22.61 | 0.80±0.03 |
|  | 80 | 2.01±0.23 | 249.13±58.59 | 0.64±0.06 |
| BTF2 | 30 | 3.39±0.27 | 416.27±96.61 | 0.92±0.03 |
|  | 60 | 3.77±0.08 | 465.77±89.10 | 0.94±0.00 |
|  | 80 | 3.33±0.33 | 328.57±22.41 | 0.90±0.05 |
