# Supplementary material for: Enhanced biological removal of intermittent VOCs and deciphering the roles of sodium alginate and polyvinyl alcohol in biofilm formation
Source: PLoS One. 2019 May 22;14(5):e0217401. doi: 10.1371/journal.pone.0217401 (PMC6530866; doi:10.1371/journal.pone.0217401)
Supplement: S4 Table — (DOCX) [file pone.0217401.s006.docx]

S4 Table. Relative abundances of dominant phyla during the operating time in the two BTFs.

| phylum | Inoculum (%) | BTF1-30  (%) | BTF1-60  (%) | BTF1-80  (%) | BTF2-30  (%) | BTF2-60  (%) | BTF2-80  (%) |
| --- | --- | --- | --- | --- | --- | --- | --- |
| Acidobacteria | 0.09 | 55.26 | 41.84 | 60.04 | 4.14 | 6.10 | 12.10 |
| Proteobacteria | 80.76 | 18.75 | 23.96 | 10.18 | 29.58 | 41.47 | 37.39 |
| Bacteroidetes | 0.42 | 18.74 | 7.95 | 15.73 | 27.48 | 30.58 | 36.71 |
| Chloroflexi | 0.01 | 2.83 | 15.85 | 9.05 | 5.20 | 7.96 | 3.20 |
| Actinobacteria | 0.03 | 0.11 | 5.03 | 1.35 | 1.11 | 6.02 | 6.98 |
| Chlorobi | 0.00 | 2.61 | 1.18 | 1.04 | 19.59 | 3.36 | 1.17 |
| Firmicutes | 18.65 | 0.28 | 0.09 | 0.32 | 0.48 | 1.61 | 0.33 |
| Thermi | 0.00 | 0.38 | 1.65 | 0.11 | 0.64 | 0.25 | 0.36 |
| Cyanobacteria | 0.00 | 0.01 | 0.07 | 0.01 | 8.22 | 0.08 | 0.01 |
| Planctomycetes | 0.00 | 0.30 | 1.81 | 0.44 | 1.20 | 0.46 | 0.07 |
| Gemmatimonadetes | 0.01 | 0.64 | 0.29 | 0.05 | 0.06 | 0.59 | 0.54 |
| Others | 0.03 | 0.11 | 0.28 | 1.69 | 2.30 | 1.51 | 1.13 |
